# Discovering common pathogenetic processes between COVID-19 and diabetes mellitus by differential gene expression pattern analysis

**DOI:** 10.1093/bib/bbab262

**Published:** 2021-07-17

**Authors:** Md Rezanur Rahman, Tania Islam, Md Shahjaman, Md Rafiqul Islam, Salvo Danilo Lombardo, Placido Bramanti, Rosella Ciurleo, Alessia Bramanti, Andrey Tchorbanov, Francesco Fisicaro, Paolo Fagone, Ferdinando Nicoletti, Manuela Pennisi

**Affiliations:** Department of Biotechnology and Genetic Engineering, Faculty of Biological Sciences, Islamic University, Kushtia, Bangladesh; Department of Biochemistry and Biotechnology, Khwaja Yunus Ali University, Enayetpur, Sirajganj, Bangladesh; Department of Biotechnology and Genetic Engineering, Faculty of Biological Sciences, Islamic University, Kushtia, Bangladesh; Department of Statistics, Begum Rokeya University, Rangpur, Bangladesh; Faculty of Health, Institute of Health and Biomedical Innovation, Queensland University of Technology (QUT), Brisbane, Australia; Department of Pharmacy, Faculty of Biological Science and Technology, Jashore University of Science and Technology, Jashore, Bangladesh; CeMM Research Center for Molecular Medicine of the Austrian Academy of Sciences, Lazarettgasse 14, AKH BT 25.3, A-1090 Vienna, Austria; IRCCS Centro Neurolesi “Bonino-Pulejo”, Via Provinciale Palermo, Contrada Casazza, 98124 Messina, Italy; IRCCS Centro Neurolesi “Bonino-Pulejo”, Via Provinciale Palermo, Contrada Casazza, 98124 Messina, Italy; IRCCS Centro Neurolesi “Bonino-Pulejo”, Via Provinciale Palermo, Contrada Casazza, 98124 Messina, Italy; Laboratory of Experimental Immunology, Institute of Microbiology , Bulgarian Academy of Sciences, Sofia, Bulgaria; National Institute of Immunology, Sofia, Bulgaria; Department of Biomedical and Biotechnological Sciences, University of Catania, 95124 Catania CT, Italy; Department of Biomedical and Biotechnological Sciences, University of Catania, 95124 Catania CT, Italy; Department of Biomedical and Biotechnological Sciences, University of Catania, 95124 Catania CT, Italy; Department of Biomedical and Biotechnological Sciences, University of Catania, 95124 Catania CT, Italy

**Keywords:** COVID-19, diabetes mellitus, blood gene expression, transcriptional signatures, molecular pathways

## Abstract

Coronavirus disease 2019 (COVID-19) is an infectious disease caused by the newly discovered coronavirus, SARS-CoV-2. Increased severity of COVID-19 has been observed in patients with diabetes mellitus (DM). This study aimed to identify common transcriptional signatures, regulators and pathways between COVID-19 and DM. We have integrated human whole-genome transcriptomic datasets from COVID-19 and DM, followed by functional assessment with gene ontology (GO) and pathway analyses. In peripheral blood mononuclear cells (PBMCs), among the upregulated differentially expressed genes (DEGs), 32 were found to be commonly modulated in COVID-19 and type 2 diabetes (T2D), while 10 DEGs were commonly downregulated. As regards type 1 diabetes (T1D), 21 DEGs were commonly upregulated, and 29 DEGs were commonly downregulated in COVID-19 and T1D. Moreover, 35 DEGs were commonly upregulated in SARS-CoV-2 infected pancreas organoids and T2D islets, while 14 were commonly downregulated. Several GO terms were found in common between COVID-19 and DM. Prediction of the putative transcription factors involved in the upregulation of genes in COVID-19 and DM identified *RELA* to be implicated in both PBMCs and pancreas. Here, for the first time, we have characterized the biological processes and pathways commonly dysregulated in COVID-19 and DM, which could be in the next future used for the design of personalized treatment of COVID-19 patients suffering from DM as comorbidity.

## Introduction

SARS-CoV-2 is responsible for the novel coronavirus disease 2019 (COVID-19), which has become a massive threat for humanity worldwide [1, 2]. Many factors influence the outcome of the disease, such as age, diabetes, hypertension and lung disease [3].

Diabetes mellitus (DM) is a chronic metabolic disease characterized by elevated blood glucose levels, due to the lack of insulin production, the resistance to insulin signaling or both. There are two main conditions, namely type 1 DM (T1D) and type 2 DM (T2D), with pathogenetic and clinical differences. T1D is characterized by the destruction of pancreatic beta cells by T cells of the immune systems [4]. On the other hand, although sub-chronic immune-inflammatory pathways are implicated in the pathogenesis of T2D, the primary culprits in the development of the disease seem to depend on peripheral insulin resistance, which is often secondary to obesity.

The prevalence of DM has increased worldwide with changing lifestyles and rising obesity. Approximately, 90% of all cases of diabetes regards T2D, and another around 10% of cases are classified as T1D. According to the International Diabetes Federation, in 2019, around 463 million people aged between 20 and 79 years had DM [5]. People with both T1D and T2D may experience diabetes-related complications, including cardiovascular disease, kidney disease, neuropathy, blindness and lower extremity amputation [6].

As expected, patients with DM and especially those with long-lasting disease and related complications [7, 8] have been shown to exhibit a more severe course of COVID-19 than non-diabetic individuals [7, 8]. It has also been proposed that COVID-19 exposure can precipitate T1D onset [9].

The synergistic effect of hyperglycaemia and COVID-19-related hyperinflammation might predispose DM patients to increased vulnerability and lethal outcomes in SARS-CoV-2 infection [10, 11].

**Figure 1:**
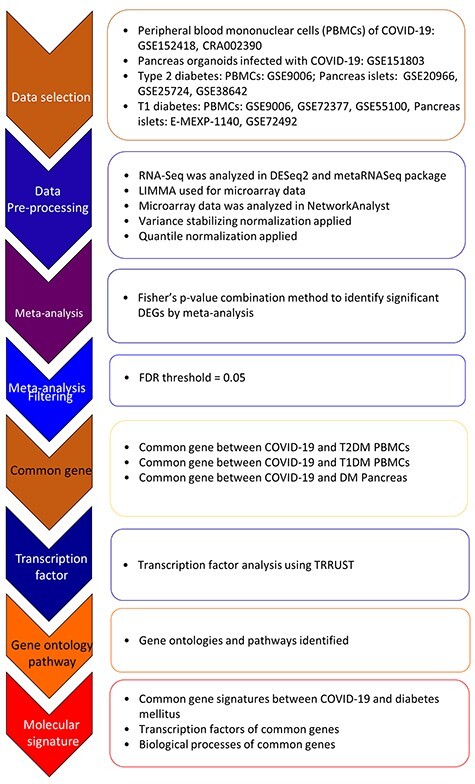
Flow chart of the analysis employed in the present study.

Overall, these observations forge the hypothesis that DM patients are more susceptible to COVID-19 infection [12]. As COVID-19 patients with DM have an elevated risk of complicated outcomes, it becomes essential to identify eventual synergistic biomolecular pathways triggered by COVID-19 and DM, which could lead to a tailored approach for the treatment of diabetic patients with COVID-19. However, the molecular alterations in common between COVID-19 with DM have not yet been investigated. The characterization of the molecular pathways is crucial to identify therapeutic targets or drug repurposing for DM patients infected with COVID-19. Recently, several studies have been profiled the transcriptomic changes occurring in COVID-19 [13–16]. However, the link between the increased vulnerability to COVID-19 in DM is still not completely clear.

This study has adopted a bioinformatic approach to identify commonly deregulated gene signatures, gene ontologies, pathways and regulators that underlie COVID-19 and DM (a summary of the study layour is presented as Figure 1).

## Materials and Methods

### Transcriptomic data acquisition

In order to identify suitable datasets generated from peripheral blood mononuclear cells (PBMCs) from COVID-19 and T2D patients, we queried the NCBI Gene Expression Omnibus (GEO) and the ArrayExpress databases. We retrieved the GSE152418 dataset generated by RNA sequencing (RNA-Seq) [17]. The dataset included 16 PBMCs samples from COVID-19 subjects and 17 healthy controls. The median age (years) of the COVID-19 patients was 56 (range: 25–94) [17]. We also obtained another PBMCs COVID-19 dataset from the Genome Sequence Archive, which included a total of six samples representing PBMCs from 3 COVID-19 and 3 healthy controls (Accession number: CRA002390) [18]. In order to retrieve datasets generated from PBMCs of T1D and T2D patients, our inclusion criteria were: (i) gene expression data, (ii) the dataset should contain case and matched control, (iii) human PBMCs samples and (iv) no drug treatment. Considering these criteria, we identified one microarray dataset (GSE9006) that included 12 T2D patients and 24 healthy controls. Finally, for T1D, we retrieved three datasets with accession number GSE9006 [19], GSE72377 and GSE55100 [20]. The GSE9006 dataset contained 81 T1D and 24 healthy control data [19]. The GSE72377 dataset contained mRNAs gene expression profile of unstimulated PBMCs from 15 T1D and 20 healthy controls (quality control data are presented as Supplementary Figure 1, see Supplementary Data available online at http://bib.oxfordjournals.org/). The GSE55100 dataset contained mRNAs gene expression profiles from T1D PBMCs from 12 cases and 10 healthy controls [20].

For the determination of the common biological processes affecting the pancreas in COVID-19 and DM, the GEO and ArrayExpress databases were also interrogated. The GSE151803 [21] dataset was chosen as it included whole-genome transcriptomic data from human pancreas organoids infected *in vitro* with SARS-CoV-2 at an MOI of 0.1 for 24 h. For the determination of the T2D pancreas signature, the GSE20966 [22], GSE25724 [23] and GSE38642 [24] datasets were obtained, which comprised a total of 25 T2D and 71 controls islet data. For T1D, the GSE72492 [25] and the E-MEXP-1140 [26] dataset were found, which included whole-genome mRNA data from a total of 24 T1D and 13 controls. The characteristics of the datasets included in this study are summarized in Table 1.

**Table 1:** The characteristics of the datasets used in the present study

Accession No.	Cell/tissue sources	Platform	Samples (Case: Control)
COVID-19 datasets			
GSE152418	PBMC	Illumina NovaSeq 6000	16:17
CRA002390	PBMC	Illumina NovaSeq platform 5000	3:3
GSE151803	Pancreas organoids	Illumina NovaSeq 6000	3:3
Type 2 DM			
GSE9006	PBMC	GPL96 [HG-U133A] Affymetrix Human Genome U133A Array	12:24
GSE20966	Pancreas islet	GPL1352 [U133_X3P] Affymetrix Human X3P Array	10:10
GSE25724	Pancreas islet	GPL96 [HG-U133A] Affymetrix Human Genome U133A Array	6:7
GSE38642	Pancreas islet	GPL6244 [HuGene-1_0-st] Affymetrix Human Gene 1.0 ST Array [transcript (gene) version]	9:54
Type 1 DM			
GSE9006	PBMC	GPL96 [HG-U133A] Affymetrix Human Genome U133A Array	81:24
GSE72377	PBMC	GPL10558 Illumina HumanHT-12 V4.0 expression beadchip	15:20
GSE55100	PBMC	GPL570 [HG-U133_Plus_2] Affymetrix Human Genome U133 Plus 2.0 Array	12:10
E-MEXP-1140	Pancreas	[HG-U133_Plus_2] Affymetrix GeneChip Human Genome U133 Plus 2.0	14:6
GSE72492	Pancreas	GPL14550 Agilent-028004 SurePrint G3 Human GE 8x60K Microarray	10:7

### Data processing and differential expression analysis

The two datasets of COVID-19 PBMCs (with accession number: GSE152418 and CRA002390) were first analyzed using the DESeq2 R package, using as statistical threshold an absolute log-fold change (logFC) ≥ 1 and Benjamini–Hochberg corrected adjusted *P*-value (false discovery rate—FDR) < 0.05. Then, we meta-analyzed these two RNA-seq datasets using Fisher’s inverse method, using the metaRNASeq R package (designed for meta-analysis of RNA transcriptomic datasets).

For the meta-analysis of the microarray datasets, we used the Network Analyst (NA) web utility tool [27]. For the normalization of the datasets, we employed variance stabilizing normalization algorithm [28], followed by the quantile normalization [29]. ComBat procedure embedded into NA was also performed to adjust study batch effect in meta-analysis. Finally, the meta-analysis of the datasets was performed using the Fisher’s *P*-value combination method.

Since we had only one T2D PBMCs gene expression dataset (GSE9006) and one for SARS-CoV-2 infected pancreas organoids (GSE151803), the significant genes were selected using the R package LIMMA (linear models for microarray data) [30], considering an FDR < 0.05.

### Functional insights into the DEGs

The enrichment analysis was performed using the widely utilized tool ‘Metascape’ [31]. Compared with other GO-based enrichment methods, it includes additional, optimized ontology databases, such as MSigDB, and clusters corresponding terminology to eliminate repetition [31]. By default, the Metascape gene functional enrichment makes use of several databases, including gene ontology (GO), KEGG, Reactome, and MSigDB databases. Metascape uses hypergeometric tests and the Benjamini–Hochberg *P*-value correction algorithm to classify all ontological parameters containing a substantially higher set of genes common to an input list than expected casually. The pairwise similarity between any two enriched terms is computed based on a Kappa-test score. The matrix of similarity is clustered hierarchically and the 0.3 threshold is applied to trim the resulting tree into different clusters. Metascape determines the most significant (lowest *P*-value) term in each cluster to depict the cluster in bar graph and heatmap. Significant GO terms were selected using as threshold a Bonferroni-corrected *P*-value <0.05.

### Transcription factor analysis

The putative transcription factors (TFs) regulating the expression of the differentially expressed genes (DEGs) were predicted via Metascape [31], using the TRRUST database, which is a manually curated database of human and mouse transcriptional regulatory networks [32, 33].

### Drug prediction analysis

The web-utility L1000FDW was employed to detect potential repurposable pharmacological agents for treating COVID-patients with DM as comorbidity. In order to rank drugs that can theoretically reverse a transcriptional signature, L1000FWD computes the similarity between the input gene expression vector and the data for LINCS-L1000. LINCS-L1000 includes the transcriptional profiles of around 50 human cell lines upon exposure to ~20 000 compounds, at different doses and times. An adjusted *P*-value (*q*-value) <0.05 has been regarded as threshold for significance.

## Results

### Analysis of PBMCs in COVID-19 and DM

#### Identification of common transcriptional signatures between COVID-19 and T2D PBMCs

**Figure 2:**
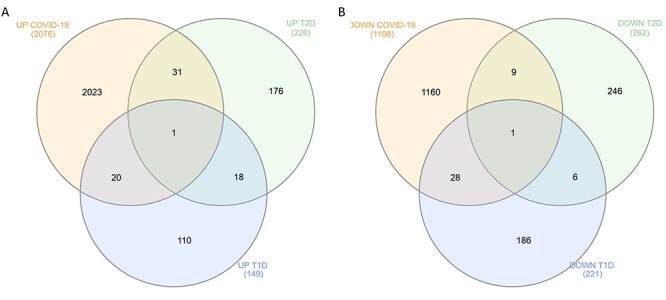
The common DEGs identified in PBMCs from COVID-19 and DM patients. (**A**) Venn diagram showing the common upregulated DEGs among COVID-19, T1D and T2D PBMCs. (**B**) Venn diagram showing the common downregulated DEGs among COVID-19, T1D and T2D PBMCs.

We obtained two PBMCs gene expression datasets from COVID-19 infected subjects and matched healthy controls (with accession numbers, GSE152418 and CRA002390) for a total of 39 samples, 19 cases and 20 healthy controls. The meta-analysis of the two datasets identified 3274 significant (FDR < 0.05) genes. The analysis of the GSE9006 dataset identified 486 DEGs (FDR < 0.05) characterizing T2D PBMCs. Among the upregulated DEGs, 32 were found to be commonly modulated in COVID-19 and T2D (*P* < 0.05) (Figure 2A), while 10 DEGs were commonly downregulated (Figure 2B).

#### Identification of common transcriptional signatures between COVID-19 and T1D PBMCs

To determine the transcriptional signature common between COVID-19 and T1D in PBMCs, we first meta-analyzed three T1D PBMCs datasets (GSE9006, GSE72377, GSE55100) comprising 162 samples of which 111 cases and 51 healthy controls. The meta-analysis identified 370 significant DEGs (FDR < 0.05). Among them, 21 were commonly upregulated (Figure 2A) and 29 DEGs commonly downregulated in COVID-19 and T1D (*P* < 0.0001) (Figure 2B). Overall, the comparative analysis of the transcriptional signatures characterizing COVID-19 and T1D PBMCs suggests the presence of commonly dysregulated genes between COVID-19 and T1D.

#### Identification of common functional GO terms in COVID-19 and DM PBMCs

**Figure 3:**
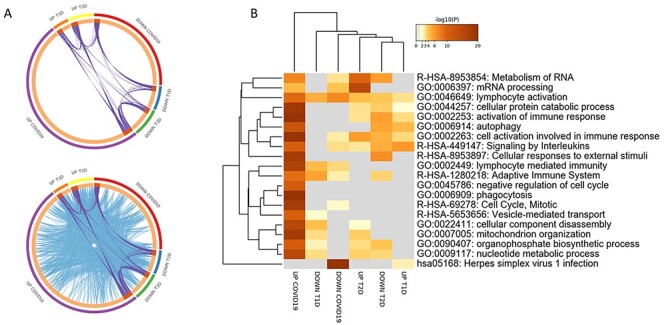
Functional enrichment analysis performed on the DEGs in COVID-19 and DM PBMCs. (**A**) Circos plot represents overlapping among the DEGs in COVID-19 and T1D and T2D PBMCs. Purple lines link the same genes that are shared by the input lists. Blue lines link the different genes that fall in the same ontology term. (**B**) Hierarchical clustering of the top 20 most enriched terms among the DEGs in COVID-19 and T1D and T2D PBMCs. The heatmap is colored by the *P*-values, and grey cells indicate the lack of significant enrichment.

**Figure 4:**
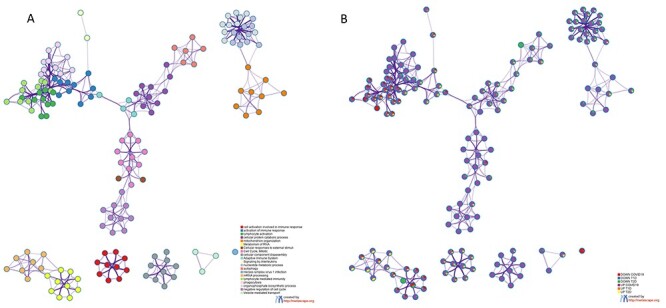
Functional analysis on the DEGs in COVID-19 and DM PBMCs. (**A**) Network showing the interconnection among the most enriched terms among the DEGs in COVID-19 and T1D and T2D PBMCs. Each term is represented by a circle node, and its color represents its cluster identity. (**B**) The same enrichment network where its nodes are displayed as pies. Each pie sector is proportional to the number of hits represented by the DEGs belonging to each gene term.

Several GO terms were found in common between COVID-19 and DM (Figure 3A, Supplementary Table 1, see Supplementary Data available online at http://bib.oxfordjournals.org/). The terms significantly enriched by the upregulated DEGs in common between COVID-19 and T2D, but not with T1D, were related to the mRNA metabolism, sub-cellular organelle organization and nucleotide synthesis (Figures 3B and 4).

The pathways that were significantly enriched by the downregulated DEGs, and in common between COVID-19 and T2D, but not in T1D were related to the mRNA metabolism and to immune responses (Figures 3B and 4). On the other hand, the autophagy process was found to be enriched among the upregulated DEGs in T1D and COVID-19, but not in T2D (Figure 3B and Figure 4).

#### Prediction of TF overlapping between COVID-19 and DM PBMCs

**Figure 5:**
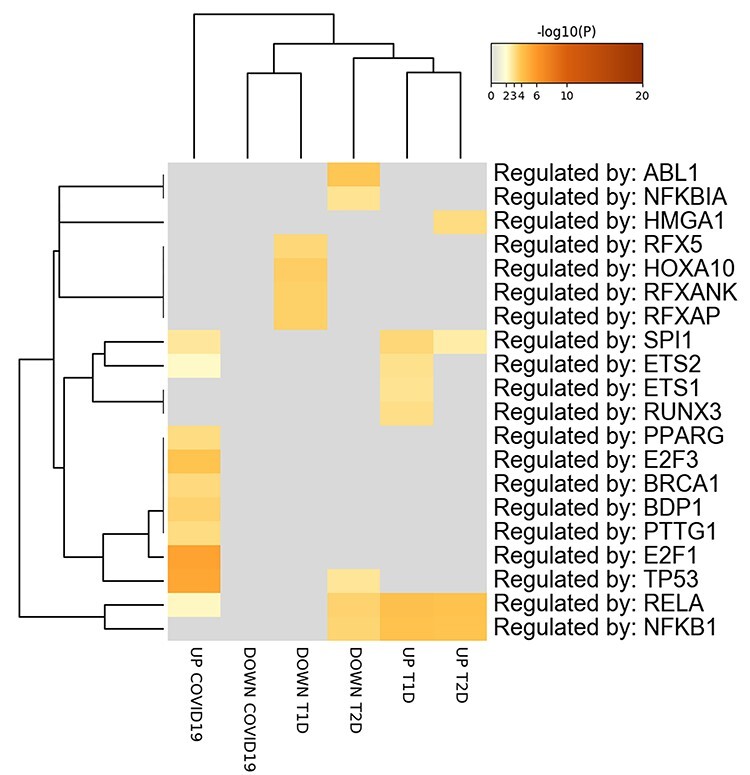
Putative TFs regulating the DEGs in PBMCs from COVID-19 and T1D and T2D patients. The TFs are visualized as a hierarchical clustering. The heatmap is colored by the *P*-values, and grey cells indicate the lack of significant enrichment.

Determination of the putative TFs involved in the regulation of the DEGs implicated in COVID-19 and DM showed that *SPI1* and *RELA* are involved in the expression of commonly upregulated genes in COVID-19, T1D and T2D. On the other hand, no significant enrichment was observed for TFs controlling the downregulated DEGs (Figure 5).

**Figure 6:**
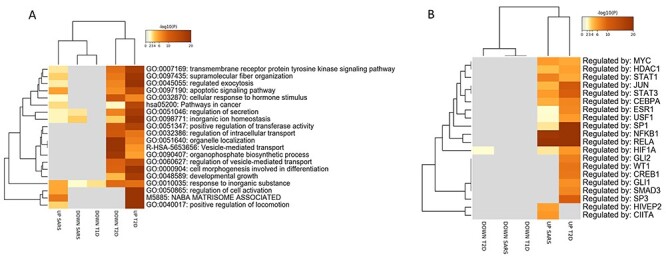
Functional analysis on the DEGs in COVID-19 and DM pancreas. (**A**) Hierarchical clustering of the top 20 most enriched terms. The heatmap is colored by the *P*-values, and grey cells indicate the lack of significant enrichment. (**B**) Putative TFs regulating the DEGs in COVID-19 and DM datasets (both type 1 and type 2) pancreas. The TFs are visualized as a hierarchical clustering. The heatmap is colored by the *P*-values, and grey cells indicate the lack of significant enrichment.

#### Drug prediction for COVID-19 patients suffering from DM

To predict drugs potentially helpful in treating COVID-19 patients with DM comorbidity, we used the L1000FWD web-based utility. For T1D, the top five drugs with the most anti-similar signature were: WAY-213613; gitoxigenin; fatostatin; BRD-K63425657 and emetin (Table 2).

**Table 2:** Top 25 drugs predicted for the use in COVID19 patients with T1D comorbidity

Drug	Similarity score	*P*-value	*q*-value	Z-score	Combined score
WAY-213613	−0.1463	1.03E−04	6.29E−01	1.68	−6.7
gitoxigenin	−0.122	7.10E−04	8.61E−01	1.84	−5.78
fatostatin	−0.122	7.47E−04	8.61E−01	1.78	−5.56
BRD-K63425657	−0.122	8.08E−04	8.61E−01	1.74	−5.37
emetine	−0.122	6.41E−04	8.61E−01	1.8	−5.76
GSK-2126458	−0.122	1.01E−03	8.61E−01	1.6	−4.79
hymecromone	−0.122	7.85E−04	8.61E−01	1.81	−5.62
BRD-K83509924	−0.0976	5.45E−03	8.61E−01	1.66	−3.76
PF-562271	−0.0976	4.91E−03	8.61E−01	1.71	−3.95
podophyllotoxin	−0.0976	5.85E−03	8.61E−01	1.65	−3.69
lestaurtinib	−0.0976	5.45E−03	8.61E−01	1.73	−3.91
BRD-K65904652	−0.0976	7.22E−03	8.61E−01	1.65	−3.53
VU-0418939-2	−0.0976	8.14E−03	8.61E−01	1.73	−3.61
digoxigenin	−0.0976	5.85E−03	8.61E−01	1.83	−4.09
sunitinib	−0.0976	5.54E−03	8.61E−01	1.73	−3.9
CFM-1571	−0.0976	5.33E−03	8.61E−01	1.87	−4.26
BRD-K59556282	−0.0976	5.81E−03	8.61E−01	1.73	−3.87
fenbendazole	−0.0976	8.37E−03	8.61E−01	1.78	−3.7
SA-1447005	−0.0976	6.18E−03	8.61E−01	1.76	−3.88
vincristine	−0.0976	6.56E−03	8.61E−01	1.67	−3.65
ALW-II-38-3	−0.0976	5.67E−03	8.61E−01	1.74	−3.91
EI-346-erlotinib-analog	−0.0976	6.08E−03	8.61E−01	1.73	−3.84
BRD-K14027855	−0.0976	6.18E−03	8.61E−01	1.76	−3.9
triciribine	−0.0976	6.61E−03	8.61E−01	1.7	−3.71
VU-0418933-1	−0.0976	5.85E−03	8.61E−01	1.75	−3.9

No drug anti-signature was found to be significantly enriched when considering the DEGs overlapping the COVID-19 and T2D PBMCs profiles (data not shown).

### Analysis of pancreas-related processes in COVID-19 and DM

#### Identification of common functional GO terms and TFs in COVID-19 and DM pancreas

Comparative analysis revealed that 35 DEGs were commonly upregulated in COVID-19 infected organoids and T2D islets, while no overlapping was found for T1D. Among the downregulated DEGs in COVID-19 infected organoids, 14 overlaped with the downregulated DEGs in T2D islets. No overlapping was instead found for T1D.

**Figure 7:**
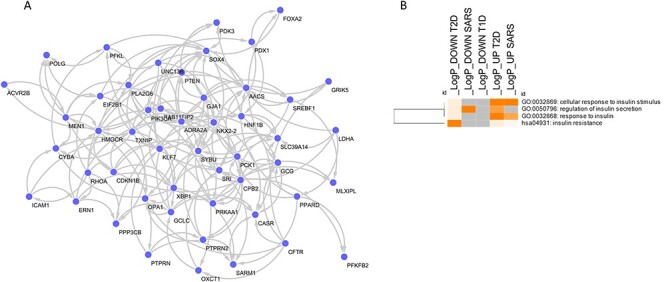
(**A**) Network constructed using the genes belonging to the ‘response to glucose’ (GO:0009749) term, enriched among the downregulated DEGs in both SARS-CoV-2 infected pancreas organoids and T2D islets. The network has been built using the GeneMania software (https://genemania.org/) and visualized in Cytoscape. (**B**) Hierarchical clustering of the enriched terms related to the insulin pathways. The heatmap is colored by the *P*-values, and grey cells indicate the lack of significant enrichment.

GO term enrichment analysis revealed several common processes characterizing both SARS-CoV-2 infection and T2D, including ‘transmembrane receptor protein tyrosine kinase signaling pathway’, ‘regulated exocytosis’ and ‘apoptotic signaling pathway. The ‘regulation of secretion’ and ‘inorganic ion homeostasis’ terms were enriched by both the up- and down-regulated DEGs characterizing COVID-19 infection and T2D (Figure 6A, Supplementary Table 2, see Supplementary Data available online at http://bib.oxfordjournals.org/). Two GO terms were commonly enriched by the downregulated DEGs characterizing both COVID-19 infection and T2D: ‘plasma membrane bounded cell projection assembly’ and ‘response to glucose’ (Figure 6, Supplementary Table 2, see Supplementary Data available online at http://bib.oxfordjournals.org/). A network showing the relationships among the genes belonging to the ‘response to glucose’ term is presented as Figure 7A. Finally, our analysis revealed a disruption of the pathways related to insulin signaling, in both T2D pancreas ans SARS-CoV-2-infected pancreas organoids (Figure 7B, Supplementary Table 2, see Supplementary Data available online at http://bib.oxfordjournals.org/).

Analysis of the putative TFs involved in the regulation of the DEGs modulated in COVID-19 and DM revealed that *MYC*, *HDAC1*, *STAT1*, *JUN*, *STAT3*, *CEBP*, *AESR1*, *USF1*, *SP1*, *NFKB1*, *RELA* and *HIF1A* were commonly involved in the expression of the upregulated DEGs in COVID-19 and T2D islet. No overlapping enrichment was instead observed T1D and neither for the downregulated DEGs in COVID-19, T1D and T2D (Figure 6B).

#### Drug prediction for COVID-19 and DM pancreas

Analysis of drugs potentially useful for the treatment of COVID-19 patients concomitantly suffering from T2D was performed using the L1000FWD web-based utility, on the 35 upregulated and 14 downregulated genes in common between COVID-19 infected pancreas organoids and T2D islets. The top three drugs with the most anti-similar signature were: vemurafenib, milnacipran and erbstatin-analog (Table 3). No drug was found to be significantly enriched when considering the DEGs overlapping the COVID-19 and T1D pancreas profiles (data not shown).

**Table 3:** Top 25 drugs predicted for the use in COVID19 patients with T2D comorbidity

Drug	Similarity score	*P*-value	*q*-value	Z-score	Combined score
vemurafenib	−0.2245	5.95E−10	5.34E−06	1.82	−16.75
milnacipran	−0.2041	1.43E−08	3.08E−05	1.76	−13.81
erbstatin-analog	−0.2041	8.79E−09	3.08E−05	1.69	−13.63
TPCA-1	−0.1837	1.30E−07	1.38E−04	1.84	−12.64
MW-STK33-2A	−0.1837	2.63E−07	1.46E−04	1.66	−10.93
cinobufagin	−0.1837	1.54E−07	1.38E−04	1.72	−11.71
dasatinib	−0.1837	3.78E−07	1.90E−04	1.6	−10.28
enzastaurin	−0.1837	2.87E−07	1.55E−04	1.69	−11.03
pifithrin-mu	−0.1837	1.83E−07	1.38E−04	1.7	−11.45
RAN-03	−0.1633	1.99E−06	5.79E−04	1.76	−10.02
BRD-K42499654	−0.1633	3.53E−06	6.41E−04	1.74	−9.48
rottlerin	−0.1633	7.69E−06	1.29E−03	1.75	−8.96
PD-98059	−0.1633	2.31E−06	5.79E−04	1.65	−9.33
XMD-132	−0.1633	2.50E−06	5.79E−04	1.7	−9.5
atracurium	−0.1633	5.05E−06	8.75E−04	1.7	−8.98
BRD-K89486464	−0.1633	2.80E−06	5.99E−04	1.75	−9.72
bosutinib	−0.1633	1.71E−06	5.79E−04	1.73	−9.98
daunorubicin	−0.1633	2.80E−06	5.99E−04	1.79	−9.95
CYT-997	−0.1633	3.22E−06	6.16E−04	1.78	−9.76
MW-STK33-1C	−0.1633	1.37E−06	5.46E−04	1.71	−10.04
BRD-A18725729	−0.1633	2.06E−06	5.79E−04	1.78	−10.11
BRD-K95976153	−0.1633	1.60E−06	5.79E−04	1.77	−10.27
BRD-K94493764	−0.1633	1.27E−06	5.46E−04	1.77	−10.45
BRD-K48654774	−0.1633	1.62E−06	5.79E−04	1.79	−10.34
amoxapine	−0.1633	3.07E−06	6.08E−04	1.69	−9.32

## Discussion

Comorbidities are associated to a higher risk of develop severe forms of COVID-19, with consequent need of mechanical ventilation and increased death rate [34]. Among the comorbidities that affect the prognosis of COVID-19 patients, DM has emerged as a potential risk factor [10]. This may in part be related to the observation that SARS-CoV-2 binds to the angiotensin-converting enzyme 2 (*ACE2*) receptors, which are expressed in metabolic organs and tissues, such as pancreatic beta cells, adipose tissue, the small intestine and the kidneys [35]. Therefore, elucidating the key genes and pathways in COVID-19 and DM is crucial to decipher the molecular association and mechanisms shared by these pathologies. The use of whole-genome transcriptomic analyses has been largely exploited by ourselves and others to study autoimmune diseases, cancer and neurodegenerative disorders [36–40], as well as to characterize potential pathogenetic mechanisms and novel therapeutic targets [41–43]. Here, we have comprehensively studied the pancreas and blood transcriptomic changes occurring in DM and COVID-19 by using integrative bioinformatics approaches. We detected 10 common down-regulated genes and 32 common upregulated genes between COVID-19 and T2D in PBMCs. Among them, it is worth noting *TSPYL4* (*Testis-specific Y-encoded-like protein 4*), for which a genetic variants has been associated with chronic pulmonary obstructive disorder [44], and *ACSL1* (*Acyl-CoA Synthetase long chain family member 1*) that encodes for the isozyme of the long-chain fatty-acid-coenzyme A ligase family. It has been shown that the inflammatory phenotype in rat model of T1D is linked to increased expression of *ACSL1*, and deletions of this gene prevent the acquisition of the inflammatory phenotype of macrophages associated with T1D [45]. Along the same lines, our study revealed altered immune response signaling pathways modulated in both COVID-19 and T2D PBMCs. In a recent study, Codo *et al.* [46] suggested that increased levels of blood glucose in T2D patients and glycolysis may promote the replication of the SARS-CoV-2 and higher production of cytokines by monocytes via mitochondrial ROS/hypoxia-inducible factor-1a-dependent pathway, which may result in T cell dysfunction and epithelial cell death. These observations suggest that metabolic inflammation in T2D patients may be favorable for viral replications and could enhance the release of cytokines.

Our results also suggested crucial pathways involved in T1D and COVID-19, such as lymphocyte activation, cellular protein catabolic process, immune response and autophagy. It has been previously shown that higher activity of lymphocytes is observed in T1D [47]; however, lymphocytes and immunoinflammatory events play a role in T2D, as well [48]. Our results suggest the ‘activation of immune response’ and ‘signaling by interleukins’ as crucial GO terms in COVID-19 and T1D, which is consistent with previous observations confirming the dysregulation of immune systems in response to viral infections [49]. The impairment of innate immunity has been linked to the increased prevalence of infections in DM patients [49]. Similarly, in an animal model, comorbid DM results in immune dysregulation and increases the severity of the diseases following infection with the other coronavirus, MERS-CoV [50].

It is also believed that metabolic inflammation compromises the immune systems in DM patients, which corroborates our results that systemic immune alterations in PBMCs could play a crucial role in both COVID-19 and T1D [51]. The impairment of the innate immunity has been also linked to the increased prevalence of infections in DM patients [49]. Moreover, the ‘cytokine storm’ occurring in COVID-19 results from uncontrolled host immune responses, which are believed to be associated with increased complications and critical conditions in COVID-19 patients. Therefore, clinical trials on antibodies/drugs that target these pathways might be conducted to identify potential use in COVID-19 patients with comorbid DM.

The two main TFs that regulate the shared DEGs between COVID-19 and DM are ‘*SPI1*’ and ‘*RELA*’. While *SPI1* is involved in the inflammatory process and modulates host immune systems [52], *RELA* is implicated in NF-kB development and regulates proliferative and inflammatory cell responses [53]. We have previously detected these two TFs as critical regulators of the DEGs identified from transcriptomic analysis of COVID-19 infected lung tissue, suggesting that these two TFs might represent both biomarkers and pharmacological targets in COVID-19 [16].

Next, we profiled the gene expression signature of pancreas organoids infected with SARS-CoV-2 to detect transcriptional alterations characterizing the response to COVID-19 infection and to compare with T1D and T2D-associated transcriptomic pancreas profiles. Our analysis identified the dysregulated process involved in the ‘regulation of secretion’, which indicates that COVID-19 alters the secretory pathways of the pancreas, likely affecting the insulin secretion and impacting the prognosis of DM patients.

Glycaemic deterioration is often observed in COVID-19 patients with DM or with impaired glucose homeostasis [54]. Accordingly, SARS-CoV-2 infection was associated with need for higher doses of insulin [54]. Moreover, although ketoacidosis typically occurs in T1D patients, it was observed that more than 75% of COVID-19 patients who developed ketoacidosis had T2D [55].

It has been proposed an association between ACE2 and glucose homeostasis. ACE2 knockout mice are more susceptible than the wild-type counterpoart to high-fat diet-induced β-cell dysfunction [56]. This observation and the expression of ACE2 in the endocrine pancreas support the notion that coronaviruses could damages the pancreatic islets, leading to hyperglycaemia [57]. Notably, it was previously shown that hyperglycaemia in SARS patients persisted for up to 3 years after recovery, indicating long-term damage to β-cells upon coronovirus infection.

In our analysis, a disruption of the regulation of insulin secretion has been observed among the DEGs characterizing COVID-19 and DM. Also, among the GO terms commonly enriched by the downregulated DEGs characterizing both COVID-19 infection and T2D, we found ‘response to glucose’. Overall, our data suggest the hypothesis that SARS-CoV-2 tropism for the β-cell could cause acute impairment of insulin secretion and/or destruction of β-cells causing deterioration of the metabolic control in people with pre-existing DM or leading to the development of new-onset DM.

Finally, we have used an anti-signature-based approach [16] that aimed to detect candidate drug molecules that could reverse the DEGs signatures identified from DM PBMCs and pancreas. Interestingly, when considering the PBMCs gene expression profiles, we only found drugs potentially reverting the T1D/COVID-19, but not the T2D/COVID-19, signature. This is likely to be ascribed to the predominant immunoinflammatory processes underlying T1D pathogenesis, characterizing the autoimmune destruction of the β cells of the endocrine pancreas, but not T2D, where insulin resistance and reduced secretion of insulin by the β cells are the main culprit of the disease [58]. On the other hand, when investigating the pancreas data, we found drugs with a signature reverting the T2D/COVID-19 profile, but none for the T1D/COVID-19 common gene expression profile. This is in line with the GO analysis, which showed prevalent metabolic alterations in COVID-19 and T2D pancreas, and not common immunoinflammatory processes. In particular, on the top three predicted drugs, we have found Vemurafenib, an FDA approved drug that can induce cellular apoptosis in melanoma cells that contained BRAF mutation via interfering B-raf/MEK/ERK pathway [59]. Also, we found Milancipran, an anti-depressant of serotonin-norepinephrin class, which is used for the treatment of psychotic disorders and fibromyalgia with dubious results [60, 61], and an erbstatin-analog, which is a EGFR inhibitor [62]. It is known that many growth factor receptors are associated with viral infections, and previous studies have suggested their repurpose for COVID-19 pandemic [63, 64]. However, despite the importance of the bioinformatics and systems biology-based analyses, the clinical validation of these findings is crucial before establishing them as biomarkers and/or interventional targets for the management of COVID-19 patients.

## Conclusions

The present study aimed at discovering gene expression signatures, TFs, and dysregulated molecular pathways, which were in common between COVID-19 and T1D and T2D. Our analysis revealed common dysregulated immune-related pathways in COVID-19 and both T1D and T2D PBMCs. Accordingly, the top predicted TFs that regulate the shared DEGs between COVID-19 and DM were SPI1 and RELA, which are involved in the regulation of the infmammatory processes. However, as expected, we have observed different DEGs shared between COVID-19 and T1D and T2D.

In addition, several pathways, including those associated to the response to glucose and insulin pathways, were enriched by the DEGs in COVID-19 and DM, as observed in the pancreas-related transcriptomic profiles. This observation supports the hypothesis that SARS-CoV-2 could directly determine an impairment of insulin secretion, with consequent diruption of the metabolic control in people already suffering from DM or leading to the development of new-onset DM.

However, it should be pointed out that there are some limitations to the present study. Indeed, although the molecular pathways commonly disregulated in COVID-19 and either of the two forms of diabetes may help to better understand the pathophysiology of the increased vulnerability of DM patients to COVID-19, the mechanisms by which DM affects COVID-19 susceptibility, severity, prognosis, mortality and long-term complications require more in-depth analysis to associate gene expression changes with respective traits. In addition, there are other major organs that are involved in the pathology of DM, which may be affected upon SARS-COV-2 infection, including kidneys, heart and blood vessels. Hence, it will be important to evaluate whether these extrapulmonary tissues are affected by pathogenetic pathways in common between COVID-19 and DM.

Notwithstanding the above-mentioned limitations, our work represents the first effort to characterize the overlapping gene expression profiles that may explain the negative prognostic association between COVID-19 and DM and, hence, may set the basis for future tailored pharmacological strategies for the better management of COVID-19 patients.

**Key Points**

- Coronavirus disease 2019 (COVID-19) is an infectious disease caused by the newly discovered coronavirus, SARS-CoV-2. Increased severity of COVID-19 has been observed in patients with diabetes mellitus (DM).
- This study implemented a bioinformatic frmaework to detect common transcriptional signatures, regulators and pathways between COVID-19 and DM.
- We have integrated human whole-genome transcriptomic datasets from COVID-19 and DM, followed by functional assessment with gene ontology and pathway analyses.
- We observed 32 differentially expressed genes (DEGs) were found to be commonly modulated in COVID-19 and type 2 diabetes (T2D), while 10 DEGs were commonly downregulated in peripheral blood mononuclear cells. As regards type 1 diabetes (T1D), 21 DEGs were commonly upregulated, and 29 DEGs commonly downregulated in COVID-19 and T1D.
- We also found 35 DEGs were commonly upregulated in SARS-CoV-2 infected pancreas organoids and T2D islets.
- Here, for the first time, we have characterized the biological processes and pathways commonly dysregulated in COVID-19 and DM, which could be in the next future used for the design of personalized treatment of COVID-19 patients suffering of DM as comorbidity.

## Supplementary Material

Suppl_Figure_1_bbab262

Suppl_Table_1_bbab262

Suppl_Table_2_bbab262

## Data Availability

All data here analyzed are publicly available on NCBI Gene Expression Omnibus (GEO) and the ArrayExpress databases.

## Notes

**Md. Rezanur Rahman** is a Lecturer (on study leave) at Khwaja Yunus Ali University, Bangladesh. Currently, he is pursuing PhD at the University of Queensland, Australia. He is a Biotechnologist and Bioinformatician by training. He received the MSc and the BSc in biotechnology and genetic engineering with outstanding academic results holding the First Position in the Faculty in both degrees from Islamic University, Bangladesh. For the highest CGPA in the Faculty, he received the most prestigious award ‘Prime Minister Gold Medal 2017’ from the Prime Minister of Bangladesh. He has published many research articles in internationally reputed journals. His research focuses on understanding the molecular mechanism of complex diseases utilizing systems biology and bioinformatics.

**Tania Islam** received her BSc (Hons) and MSc in biotechnology and genetic engineering with outstanding CGPA holding second merit position from Islamic University, Bangladesh. She is now working as Research Assistant at the Bioinformatics Lab of Biotechnology and Genetic Engineering Department from Islamic University, Bangladesh. She is interested in bioinformatics multi-omics research to decipher the molecular mechanism of complex disease.

**Md. Shahjaman** has been working as an Associate Professor at the Department of Statistics, Begum Rokeya University, Rangpur, Bangladesh, since 2010. He completed his PhD in bioinformatics in 2018 from the University of Rajshahi, Bangladesh. He completed his BSc (honors) and MSc in statistics from the same institution. His research interest lies in theoretical and computational statistics, robust statistics, supervised and unsupervised machine learning, statistical inference, data science and deep learning with their application to big biological datasets such as biomarker identification from transcriptomics, metabolomics and other OMICS datasets.

**Md. Rafiqul Islam** is an Assistant Professor at the Department of Pharmacy, Jashore University of Science and Technology, Bangladesh. He has completed the B.Pharm and the M.Pharm from the University of Rajshahi, Bangladesh. Currently, he is pursuing PhD at the Queensland University of Technology, Australia. He is interested in genetics, genomics and bioinformatics research.

**Salvo Danilo Lombardo** graduated in medicine and surgery at the University of Catania, Italy (110/110 cum laude). He is currently a PhD student at the Menche’s Lab at CeMM (Research Center for Molecular Medicine of the Austrian Academy of Sciences).

**Placido Bramanti** graduated in medicine and surgery at the University of Messina (1974), specialized in neurology (1978); he is a Full Professor of applied medical technical sciences at the Department of Biomedical Sciences and Morphological and Functional Imagery of the same university and, since 2006, a Scientific Director of the IRCCS Centro Neurolesi ‘Bonino-Pulejo’ of Messina. He is coauthor of over 800 publications, numerous books, book chapters and patents.

**Rosella Ciurleo** graduated with honors in pharmaceutical chemistry and technology at the University of Messina, and then obtained a PhD in pharmaceutical sciences in 2009. Since 2009, she has been working at the IRCCS Centro Neurolesi Bonino-Pulejo. She is a PI of a research project sponsored by the Ministry of Health, which concerns the clinical application of Spectroscopic Magnetic Resonance, and the Head of the Technical-Scientific Secretariat of the Ethics Committee. She is in charge of the management, control and verification of the entire process of pharmacological and non-pharmacological clinical trials. She is the Co-PI of a project sponsored by the European Community (WP7) .

**Alessia Bramanti** graduated at the University of Messina, where she also obtained her PhD. She is employed at the IRCCS Centro Neurolesi ‘Bonino-Pulejo’, Messina, Italy. She is a co-author of more than 150 international peer-reviewed jurnals, reaching an HI of 26.

**Andrey Tchorbanov** is an Associate Professor. He has a renowed international experience obtained in Utrecht (Nederland), Taipei (Taiwan) and Budapest (Hungary). He is a group leader of projects financed by the National Science Fund of Bulgaria (L1304, TK-317, VU-L-306, VUH – 11, DO 02–312, TK-X-1611, DTK 02/19, DTK 02/78, DDVU 02/34), as well as of projects in the framework of bilateral agreements between Bulgarian Academy of Sciences and National Scientific Council (Taiwan) and Hungarian Academy of Sciences (Hungary). Dr Tchorbanov is an expert in constructing hybrid antibody molecules by protein and genetic engineering, humanized SCID and other murine models and *in vitro* immunological studies. He holds a patent N108155 ‘A tool for selective suppression of pathological DNA-specific B cells’.

**Francesco Fisicaro** graduated in medicine and surgery at the University of Catania and then obtained his Residency in Neurology. He is currently a PhD student in basic and applied biomedical sciences at the University of catania, Italy.

**Paolo Fagone** is an Assistant Professor at the Department of Biomedical and Biotechnologucal Sciences at the University of Catania, Italy. He obtained his MS degree in health biology at the University of Catania in 2006 (110/110 um laude). Afterwards, he completed his Residency training in Microbiology and Virology. He has been a Research Fellow at the Stellar-Chance Laboratories at the University of Pennsylvania, Philadelphia, for 2 years, after which he attended the School of Medicine of the University of Catania and graduated in medicine and surgery in 2018 (110/110 cum laude). He has published more than 100 papers in peer-reviewed journals and has reached an H-index of 27.

**Ferdinando Nicoletti** is a Full Professor of general pathology at the Department of Biomedical and Biotechnological Sciences at the University of Catania, Italy. He graduated in medicine and surgery at the University of Catania and obtained his Residency in Allergology and Clinical Immunology at the Institute of Internal Medicine, Infectious Diseases and Immunopathology at the University of Milan in 1990. Ferdinando Nicoletti has been working in the field of cellular and molecular immunology, immunopharmacology, toxicology and experimental oncology. He has published more than 350 papers in peer-reviewed journals and has reached an H-index (google scholar) of 66. Prof. Nicoletti serves in the Editorial Board of several international journals including *Antioxidants*, *Antioxidant and Redox Signaling*, *Basic and Clinical Pharmacology and Toxicology*, *European Journal of Pharmacology*, *International Journal of Molecular Medicine* and *Journal of Clinical Medicine*. Ferdinando Nicoletti is inventor of 5 patents (granted) and has cofounded 5 biotech companies, namely Ganial Immunotherapeutics (Delaware, USA), OncoNOx ApS (Copenhagen, Denmark), Aura Biopharm (Oslo, Norway), Roxiant (Copenhagen, Denmark) and Cresence (Oslo, Norway).

**Manuela Pennisi** graduated in medicine and surgery (110/110 cum laude) at the University of Catania in 2004. In 2009, she obtained the Residency in Neurology (70/70 cum laude). She obtained the PhD in neurobiology in 2014. From 2018, she is an Assistant Professor at the Department of Biomedical and Biotechnological Sciences, University of Catania, Italy.
