# Supplementary figures and images for: Discovering common pathogenetic processes between COVID-19 and diabetes mellitus by differential gene expression pattern analysis
Source: Brief Bioinform. 2021 Jul 17;22(6):bbab262. doi: 10.1093/bib/bbab262 (PMC8344483; doi:10.1093/bib/bbab262)

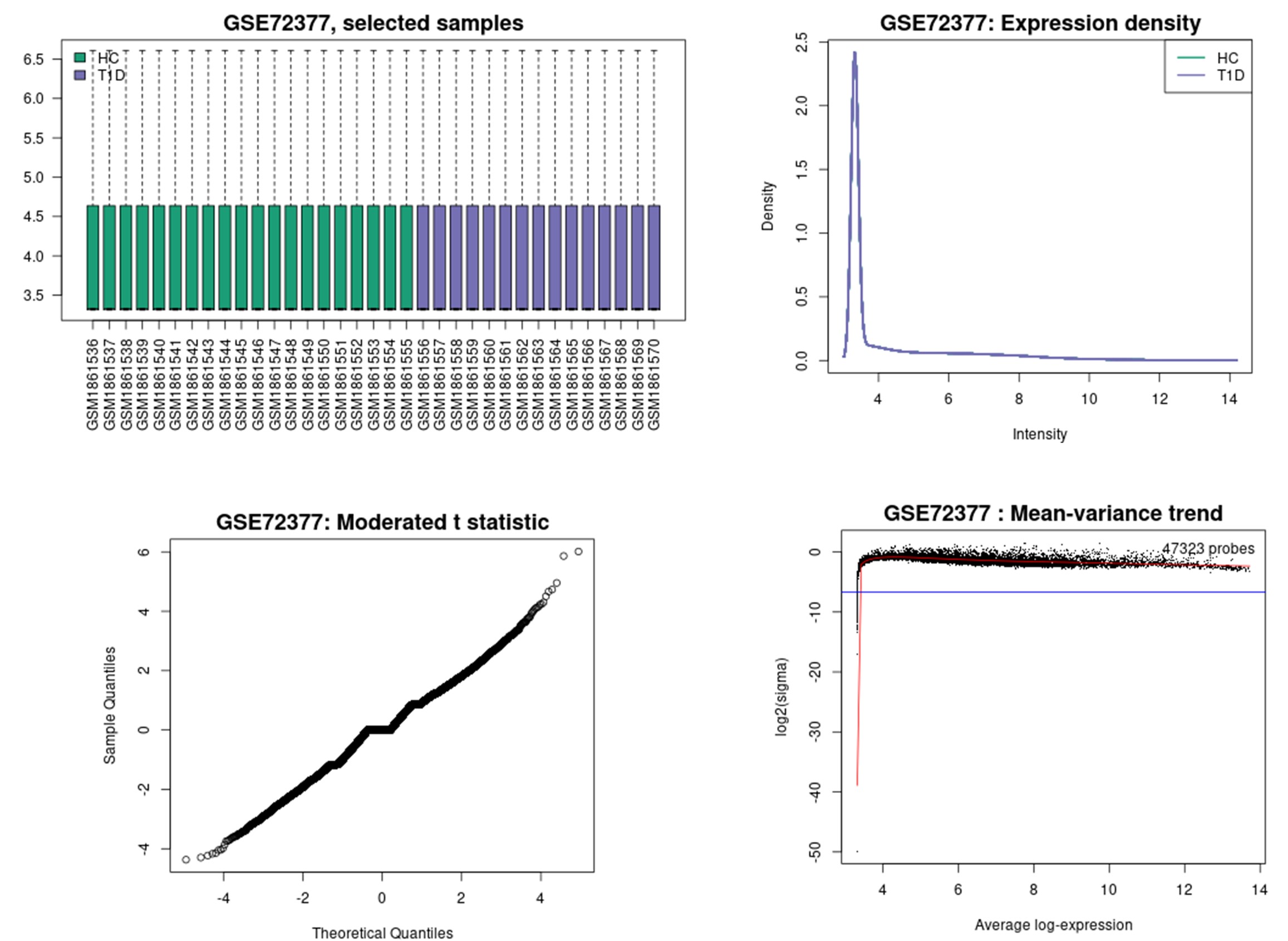

Supplement: Suppl_Figure_1_bbab262 [file suppl_figure_1_bbab262.jpeg]
